# Supplementary material for: Pasteurized Akkermansia muciniphila Ameliorate the LPS-Induced Intestinal Barrier Dysfunction via Modulating AMPK and NF-κB through TLR2 in Caco-2 Cells
Source: Nutrients. 2022 Feb 11;14(4):764. doi: 10.3390/nu14040764 (PMC8879293; doi:10.3390/nu14040764)
Supplement: Supplementary file 1 [file nutrients-14-00764-s001.zip › nutrients-1563885-supplementary.pdf]

Supporting information

Table S1. Real time-PCR primers were used in this study.

Accession	Target	Primer sequence (5′-3′)a	Product size (bp)
NM_001256799	GAPDH	F: GGAGCGAGATCCCTCCAAAAT<br>R: GGCTGTTGTCATACTTCTCATGG	196
NM_002538	occludin	F: CCTTCACCCCCATCTGACTAT<br>R: CTTTGACCTTCCTGCTCTTCC	249
NM_003257.3	ZO-1	F: GGATGTTTATCGTCGCATTGTA<br>R: AAGAGCCCAGTTTTCCATTGTA	158
NM_004817.3	ZO-2	F: CGTTGCTGGTAATGAAACTCCT<br>R: ATCTTGCTCCTCACTGCTCTCT	154
NM_021101.4	claudin1	F: AGAAGATGAGGATGGCTGTCA<br>R: TTGGTGTTGGGTAAGAGGTTG	249
NM_020384.3	claudin2	F: TTCTTCCCTGTTCTCCCTGAT<br>R: CCCCTGGTTCTTCACACATAC	198
NM_001305.3	claudin4	F: TATGGATGAACTGCGTGGTG<br>R: CACGATGATGCTGATGATGAC	122
NM_003263.4	TLR1	F: CCAAATGGAACAGACAAGCAGG<br>R: ATGAAGACCCTGGCCACAAA	116
NM_001318789.2	TLR2	F: GGTTCAAGCCCCTTTCTTCT<br>R: TTCCCACTCTCAGGATTTGC	117
NM_003266.4	TLR4	F: CCAGGATGAGGACTGGGTAA<br>R: CCTTTCGGCTTTTATGGAAA	152
NM_000594.4	TNF-α	F: AGCCCATGTTGTAGCAAACC<br>R: GGAAGACCCCTCCCAGATAG	335
NM_000575.4	IL-1α	F: GGTGTTGCCTGCTGCCTTCC<br>R: GTTCTGAAGAGGTGAGTGGCTGTC	185
NM_000576.2	IL-1β	F: GCGGCATCCAGCTACGAATCTC<br>R: AACCAGCATCTTCCTCAGCTTGTC	101
NM_000600.5	IL-6	F: AGGCTGCATGGATCAATCTGTGTC<br>R: CTTCCTCTGAGTCATTGGCGATGG	95
NM_000584.4	IL-8	F: TTTTGCCAAGGAGTGCTAAAGA<br>R: AACCCTCTGCACCCAGTTTTC	194
NM_000660.6	TGF-β	F: AGCAACAATTCCTGGCGATACCTC<br>R: TCAACCACTGCCGCACAACTC	103
NM_000572.3	IL-10	F: GCCAAGCCTTGTCTGAGATGATCC<br>R: GCTCCACGGCCTTGCTCTTG	93

[a] F and R indicated forward and reverse primers, respectively.

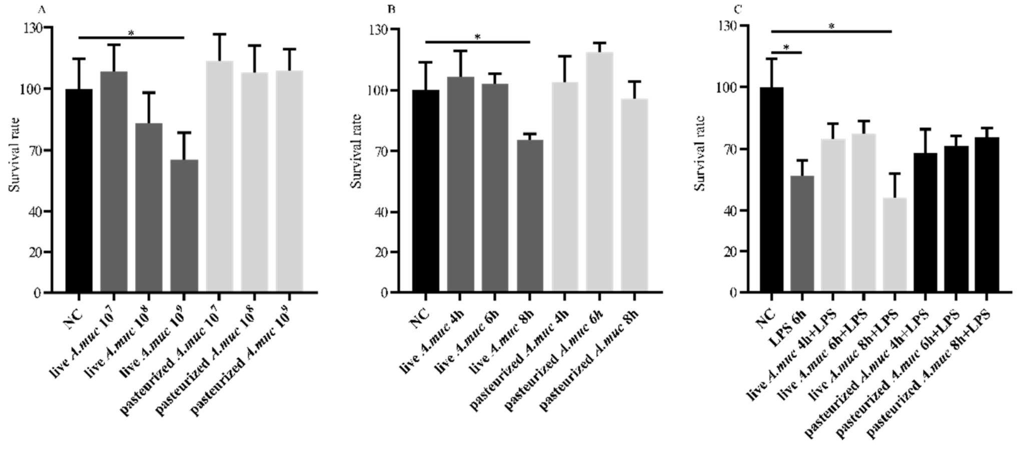

Figure S1. The survival rate of Caco-2 cell viability co-culture with (A) 1 × 10^7 CFU, 1 × 10^8 CFU and 1 × 10^9 CFU *A. muciniphila* for 6 h, (B) 1×10^7 CFU *A. muciniphila* for 4, 6, 8 h, and (C) 1×10^7 CFU *A. muciniphila* for 4, 6, and 8 h after stimulation by LPS (5 μg/mL). *$p < 0.05$, compared with respective control. Data are expressed as mean ± S.D. ($n = 6$).
